# Clinical use, efficacy, and durability of maraviroc for antiretroviral therapy in routine care: A European survey

**DOI:** 10.1371/journal.pone.0225381

**Published:** 2019-11-21

**Authors:** Andrea De Luca, Patrizio Pezzotti, Charles Boucher, Matthias Döring, Francesca Incardona, Rolf Kaiser, Thomas Lengauer, Nico Pfeifer, Eugen Schülter, Anne-Mieke Vandamme, Maurizio Zazzi, Anna Maria Geretti, for the EucoHIV Study Group

**Affiliations:** 1 Department of Medical Biotechnologies, University of Siena, Siena, Italy; 2 Unità Operativa Complessa Malattie Infettive, Azienda Ospedaliera Universitaria Senese, Siena, Italy; 3 Department of Infectious Diseases, Istituto Superiore di Sanità, Rome, Italy; 4 Department of Viroscience, Erasmus Medical Center, Rotterdam, Netherlands; 5 Max Planck Institute for Informatics, Saarland Informatics Campus, Saarbrücken, Germany; 6 EuResist Network, Rome, Italy; 7 InformaPRO, Rome, Italy; 8 Institute of Virology, University of Cologne, Cologne, Germany; 9 Department of Computer Science, University of Tübingen, Tübingen, Germany; 10 Department of Microbiology, Immunology and Transplantation, KU Leuven, Rega Institute for Medical Research, Clinical and Epidemiological Virology, Leuven, Belgium; 11 Center for Global Health and Tropical Medicine, Unidade de Microbiologia, Instituto de Higiene e Medicina Tropical, Universidade Nova de Lisboa, Lisbon, Portugal; 12 Institute of Infection and Global Health, University of Liverpool, Liverpool, England, United Kingdom; Consejo Superior de Investigaciones Cientificas, SPAIN

## Abstract

**Objectives:**

The study aimed to survey maraviroc use and assess effectiveness and durability of maraviroc-containing antiretroviral treatment (ART) in routine practice across Europe.

**Methods:**

Data were retrieved from 26 cohorts in 8 countries comprising adults who started maraviroc in 2005–2016 and had ≥1 follow-up visit. Available V3 sequences were re-analysed centrally for tropism determination by geno2pheno[coreceptor]_._ Treatment failure (TF) was defined as either virological failure (viral load >50 copies/mL) or maraviroc discontinuation for any reason over 48 weeks. Predictors of TF were explored by logistic regression analysis. Time to maraviroc discontinuation was estimated by Kaplan-Meier survival analysis.

**Results:**

At maraviroc initiation (baseline), among 1,381 patients, 67.1% had experienced ≥3 ART classes and 45.6% had a viral load <50 copies/mL. Maraviroc was occasionally added to the existing regimen as a single agent (7.3%) but it was more commonly introduced alongside other new agents, and was often (70.4%) used with protease inhibitors. Accompanying drugs comprised 1 (40.2%), 2 (48.6%) or ≥3 (11.2%) ART classes. Among 1,273 patients with available tropism data, 17.6% showed non-R5 virus. Non-standard maraviroc use also comprised reported once daily dosing (20.0%) and a total daily dose of 150mg (12.1%). Over 48 weeks, 41.4% of patients met the definition of TF, although the 1-year estimated retention on maraviroc was 82.1% (95% confidence interval 79.9–84.2). Among 1,010 subjects on maraviroc at week 48, the viral load was >50 copies/mL in 19.9% and >200 copies/mL in 10.7%. Independent predictors of TF comprised a low nadir CD4 count, a detectable baseline viral load, previous PI experience, non-R5 tropism, having ≥3 active drugs in the accompanying regimen, and a more recent calendar year of maraviroc initiation.

**Conclusions:**

This study reports on the largest observation cohort of patients who started maraviroc across 8 European countries. In this overall highly treatment-experienced population, with a small but appreciable subset that received maraviroc outside of standard treatment guidelines, maraviroc was safe and reasonably effective, with relatively low rates of discontinuation over 48 weeks and only 2 cases of serum transaminase elevations reported as reasons for discontinuation.

## Introduction

Among antiretrovirals approved for the treatment of HIV-1 infection, maraviroc and the recently approved ibalizumab are unique in targeting a host protein rather than a viral enzyme. By engaging the transmembrane helices of the CC chemokine receptor 5 (CCR5), maraviroc disrupts the geometry of the multi-point interaction between the second extracellular loop of CCR5 and the V3 loop of HIV-1 glycoprotein (gp) 120, allosterically preventing R5 virus from binding. As a result of the targeted mode of action, a tropism test demonstrating the presence of CCR5-tropic (R5) virus is a prerequisite for maraviroc use. During clinical development, tropism was determined with the Trofile phenotypic test provided by Monogram Biosciences (CA, USA). Subsequently, largely retrospective validation studies established genotypic tropism testing as an alternative, based on the analysis of the gp120 V3 sequence through an automated predictive algorithm, most commonly the geno2pheno[coreceptor] system [1]. Comparative evaluations demonstrated a good albeit not perfect technical correlation between phenotypic and genotypic tropism testing [2–4]. Importantly, genotypic tropism testing was found to predict virological outcome within both retrospective evaluations of clinical trial data and in observational cohorts [2,5–7].

The efficacy and safety of maraviroc have been demonstrated in phase 3 clinical trials of both antiretroviral treatment (ART)-naïve and ART-experienced subjects with R5 virus [8–12]. While in the US maraviroc is approved for both indications, in Europe it is approved only for the treatment of ART-experienced subjects. In clinical practice, however, maraviroc has been used in a variety of clinical scenarios [13–18].

With the aim of gaining a comprehensive understanding of maraviroc use, this multicentre observational cohort study surveyed reasons for starting maraviroc and measured the efficacy and durability of maraviroc-containing regimens across Europe. A secondary objective was to determine the performance of genotypic tropism testing in relation to virological outcomes.

## Patients and methods

### Study population and data collection

Subjects eligible for inclusion were HIV-1 positive adults who started their first maraviroc-containing ART regimen between January 2005 and December 2013 within routine clinical practice, and had ≥1 follow-up visit after receiving ≥1 dose of maraviroc. Fully anonymised data available from individual electronic databases and clinical cohorts were collected as part of a formal European audit of clinical service; no ethics permission was required, and the audit was registered with the Royal Liverpool University Hospital in the United Kingdom. A total of 26 clinical cohorts in 8 European countries participated. Each centre adhered to local research governance regulations concerning the collection and analysis of routinely collected, anonymised clinical data for audit purposes. Data submission to the central audit repository occurred through a structured case record form that collected simple demographic data (age, gender, ethnicity), current and nadir CD4 cell count, CD4 cell count and plasma HIV-1 RNA load at the start of maraviroc (baseline, measured within 6 weeks prior to starting maraviroc) and during follow-up, ART history (number and classes of antiretrovirals received prior to baseline, calendar year of first maraviroc use, reasons for starting and stopping maraviroc, and antiretrovirals used together with maraviroc), results of drug resistance and tropism testing performed at any time before and after starting maraviroc, and HIV-1 subtype if known. We used objective measures from the database in order to classify the context of maraviroc use. ART-naïve patients were classified on the objectively verified absence of previous ART use. In ART-experienced individuals, treatment was classified as “ART switch” when one or more components of the existing regimen were changed alongside the introduction of maraviroc, and as “ART intensification” when maraviroc was added as a single agent to the existing regimen. These treatment categories were subdivided based on suppressed or detectable viral load (i.e., HIV-1 RNA either < or ≥ 50 copies/mL) prior to maraviroc initiation.

### Viral tropism and drug susceptibility

Results of genotypic tropism testing were verified by a centralised re-analysis of gp120 V3 sequences using geno2pheno[coreceptor] version 2.5 [1] with a false positive rate (FPR) defining R5-tropic virus set at >10% in case of triplicate sequences or >20% in case less than three sequences were available, as per published European guidelines [19]. Centres using phenotypic testing submitted results obtained with the Monogram Trofile or Enhanced Sensitivity Trofile assay. A valid baseline tropism assay result was measured using plasma collected within 90 days prior to starting maraviroc in viraemic patients. Older results obtained from either plasma, whole blood or peripheral blood mononuclear cells (PBMC) where accepted provided the patient had not experienced viraemia between the tropism test and the start of maraviroc, as per European guidelines [19]. The Genotypic Susceptibility Score (GSS) of the regimen accompanying maraviroc was derived from protease, reverse transcriptase, and integrase sequences using the Stanford’s HIVdb 8.1 algorithm (www.hivdb.stanford.edu), whereby each drug in the regimen was assigned a susceptibility score based on the estimated levels of drug resistance: susceptible and potential low-level resistance = 1; low-level and intermediate resistance = 0.5; and high-level resistance = 0. For enfuvirtide, gp41 sequences were interpreted using HIV GRADE (http://www.hiv-grade.de/grade/deployed/grade.pl?program=hivalg). In the absence of an integrase or gp41 sequence, integrase inhibitors and enfuvirtide were assumed to be fully active in case of first use or previous use without failure (score = 1) and not active (score = 0) in case of previous use with documented failure. HIV-1 subtypes were assigned based on all *pol* sequences using the Rega subtyping tool 3.0 (http://dbpartners.stanford.edu:8080/RegaSubtyping/stanford-hiv/typingtool) and the Comet algorithm (https://www.ncbi.nlm.nih.gov/pubmed/25120265). In case of discordant results between the two algorithms, the subtype was set as undetermined.

### Treatment outcomes for maraviroc-containing regimens and statistical analysis

The date of maraviroc initiation was considered the baseline for the study. Treatment outcomes were measured over 48 weeks (allowing a window of +/- 6 weeks). Treatment failure (TF) was defined by either virological failure (VF, viral load >50 copies/mL while on maraviroc) or maraviroc discontinuation prior to week 48 for any cause. The VF analysis comprised patients who remained on maraviroc at week 48. Time to maraviroc discontinuation was estimated by survival analysis using the Kaplan-Meier method; where data were available past week 48, these were retained in the analysis. Predictors of TF and VF were analysed by univariate and multivariable logistic regression. All p-values were calculated by a univariate mixed logistic model taking into account the clustering effect of the clinical centre. In the multivariable models, a backward stepwise selection method was employed starting from a model with all variables showing an association with the outcome of interest with a p-value ≤ 0.2 according to the univariate analysis. These models employed a stepwise exclusion criterion of variables with p-values >0.2, except for viral tropism that was manually included in all models. In the final multivariable models, all variables retained by the selection method were simultaneously adjusted. Statistical analysis was performed using the STATA version 13.0 software package.

## Results

### Study population at baseline

Data were retrieved from 26 clinical centres in 8 countries totalling 1,381 patients, and comprising 538 from the United Kingdom and Ireland, 291 from Italy, 259 from Germany, 206 from Spain, 43 from France, 30 from Belgium, and 14 from Luxemburg. The characteristics of the study population at the time of starting maraviroc (baseline) are summarized in Table 1. Most patients were males of white ethnicity (718/1,381, 52%), 754/1,381 (54.6%) with a nadir CD4 count <200 cells/mm^3^, and 782/1,381 (56.6%) with a current CD4 cell count ≥350 cells/mm^3^. Almost half (630/1,381, 45.6%) had a suppressed viral load (<50 copies/mL). The overall cohort was heavily ART-experienced, with 718/1,381 (52.0%) having received ≥7 antiretroviral drugs and about two-thirds (926/1,381, 67.1%) having experienced ≥3 drug classes including protease inhibitors (PIs) in 1213/1,381 (87.8%) and integrase inhibitors in 651/1,381 (47.1%).

**Table 1:** Characteristics of the study population at the start of maraviroc (n = 1,381).

Characteristic			
**Age, median years (IQR)**		46	41–52
**Males, n (%)**		1,052	76.7%
**Ethnicity, n (%)**	White Caucasian	718	52.0%
	Black African	231	16.7%
	Asian	18	1.3%
	Other/Unknown	414	30.0%
**Nadir CD4 count** **(cells/mm**^**3**^**)**	<50	285	20.6%
	50–199	469	34.0%
	200–349	290	21.0%
	≥350	148	10.7%
	NA	189	13.7%
**Baseline CD4 count (cells/mm**^**3**^**)**	<50	51	3.7%
	50–199	236	17.1%
	200–349	257	18.6%
	≥350	782	56.6%
	NA	55	4.0%
**HIV-1 RNA** **(copies/mL)**	<50	630	45.6%
	50–199	147	10.6%
	200–4,999	253	18.3%
	5,000–99,999	189	13.7%
	≥100,000	120	8.7%
	NA	42	3.0%
**Prior antiretrovirals, n (%)**	<3	268	19.4%
	3–6	341	24.7%
	7–10	349	25.3%
	≥10	369	26.7%
	NA	54	3.9%
**Prior drug classes, n (%)**	<3	455	32.9%
	3	482	34.9%
	≥3	444	32.2%
**PI experienced, n (%)**		1,213	87.8%
**NRTI experienced, n (%)**		1,249	90.4%
**NNRTI experienced, n (%)**		846	61.3%
**InSTI experienced, n (%)**		651	47.1%
**Enfuvirtide experienced, n (%)**		84	6.1%
**Calendar year** **of maraviroc start**	2005–2008	249	18.0%
	2009–2010	496	35.9%
	2011	311	22.5%
	2012–2016	325	23.5%

Abbreviations: ART, antiretroviral treatment; NA, not available; PI, protease inhibitor; NRTI, nucleos(t)ide reverse transcriptase inhibitor; NNRTI, non-nucleoside reverse transcriptase inhibitor; InSTI, integrase strand-transfer inhibitor.

### Viral tropism

Tropism assay results were available for 1,273 (92.3%) participants: 1,075 through genotypic testing and 189 through a phenotypic assay (Trofile in 118, Enhanced Sensitivity Trofile in 58, unspecified in 13). In 9 cases the type of assay was not specified and no V3 sequences were available for re-analysis. Both phenotypic and genotypic tropism assay results were available in 14 cases, with only one discordant case where the phenotypic result was considered. Tropism testing was performed using plasma HIV-1 RNA in 696 cases and HIV-1 DNA (from whole blood or PBMC) in 561; in 16 cases the source was not specified. The tropism result was R5 in 1,049/1,273 (82.4%) and non-R5 in 224/1,273 (17.6%). The FPR results were available for 1,036/1,075 genotypic tropism assays and comprised: <10% in 104 cases, 10% to 20% in 133 cases, 20% to 40% in 249 and ≥40% in 550 cases. The median FPR value (IQR) was 50.5 (33.9–78.1) for results assigned as R5 and 10.4 (4.6–15.5) for those assigned as non-R5.

### GSS and HIV-1 subtypes

The GSS of the regimen accompanying maraviroc was available for 661/1,381 (47.9%) subjects, including 393/709 (55.4%) subjects that started maraviroc with HIV-1 RNA ≥50 copies/mL. The GSS was <1 in 83/661 (12.6%), 1 in 254/661 (38.4%), 2 in 228/661 (34.5%), and ≥3 in 96/661 (14.5%). The viral subtype was available in 794/1,381 patients (57.5%): most carried subtype B (606/794, 76.3%), followed by C (36, 4.5%), A (33, 4.2%), CRF02_AG (19, 2.4%), G (12, 1.5%), others (43, 5.4%), and unassigned subtype (45, 5.7%).

### Patterns of maraviroc use

Clinician-reported reasons for maraviroc initiation were available in 1,297/1,381 (93.9%) patients. Most patients that started maraviroc were described as treatment experienced. Among the reported reasons, VF accounted for 547/1,297 (39.6%) initiations, whereas a change of a virologically suppressive regimen was reported in 465/1,297 (35.9%) as a result of toxicity (374, 27.1%) or other reasons (91, 6.6%). Intensification of suppressive ART was described in 104/1,297 (8.0%) subjects and the reasons comprised low CD4 cell counts (56, 4.1%) and miscellaneous other reasons including aiming to improve ART activity in the central nervous system (CNS). Maraviroc was started in ART-naïve subjects in 49/1,297 (3.5%) cases. For the remaining 132/1,297 (9.6%) subjects, the reason for maraviroc initiation was not reported. For analytic purposes, the context of maraviroc initiation was classified using objective measures (Table 2). Among the 1,381 patients with an objectively classifiable reason for maraviroc initiation, 882 (63.9%) switched ART regimen (i.e., added maraviroc while stopping and/or adding at least another agent), comprising 441 (31.9%) with a detectable viral load, 409 (29.6%) with an undetectable viral load and 32 (2.3%) with unknown viral load. The existing ART regimen was intensified with maraviroc as a single add-on agent in 101/1,381 (7.3%) subjects, comprising 50 (3.6%) with a detectable viral load, 50 (3.6%) with an undetectable viral load, and 1 (0.1%) with unknown viral load. ART-naïve maraviroc use accounted for 46/1,381 (3.3%) cases.

**Table 2:** Context of maraviroc use.

ART status	HIV-1 RNA (copies/mL)	n	% of all cases
**Starting ART from naive**	>50	46	3.3%
**Switching ART regimen**	<50	409	29.6%
	≥50	441	31.9%
	NA	32	2.3%
**Intensification of ART regimen**	<50	50	3.6%
	≥50	50	3.6%
	NA	1	0.1%
**Unknown**	<50	169	12.2%
	≥50	175	12.7%
	NA	8	0.6%

Abbreviations: ART, antiretroviral treatment; NA, not available

The most frequently employed total daily dose of maraviroc was reported as 300mg (733/1,381, 53.1%), followed by 600mg (228/1,381, 16.5%), 150mg (167/1,381, 12.1%) and 1,200mg (31/1,381, 2.2%); the dose was not reported in 222/1,381 (16.1%) of cases. The dosing schedule was reported as twice daily in 661/1,381 (47.9%) subjects, once daily in 276/1,381 (20.0%), and was not reported in 444/1,381 (32.2%). The accompanying drugs belonged to 1, 2, and 3 or more classes in 518/1,381 (40.2%), 626/1,381 (45.3%) and 144/1,381 (10.4%) regimens, respectively (Table 3); 906 (70.4%) regimens included PIs.

**Table 3:** Antiretroviral treatment classes used with maraviroc.

Number of classes	Class	n	%
**1**	Any	518	37.5
	PI	333	24.1
	NRTI or NNRTI	161	11.7
	InSTI	20	1.4
	2 PIs	4	0.3
**2**	Any	626	45.3
	NRTI + PI	111	8.0
	2–3 NRTIs + PI	192	13.9
	NRTI + NNRTI	39	2.8
	1–2 NRTIs + 2 PIs	2	0.1
	Other combination	282	20.4
**3**	Any	120	8.7
	PI + NRTI + NNRTI	13	0.9
	Other combination	107	7.7
**4**	Any	24	1.7
**Not available**		93	6.7

Abbreviations: PI, protease inhibitor; NRTI, nucleos(t)ide reverse transcriptase inhibitor

NNRTI, non-nucleoside reverse transcriptase inhibitor; InSTI, integrase strand-transfer inhibitor.

### Treatment outcomes of maraviroc containing regimens

**Fig 1:**
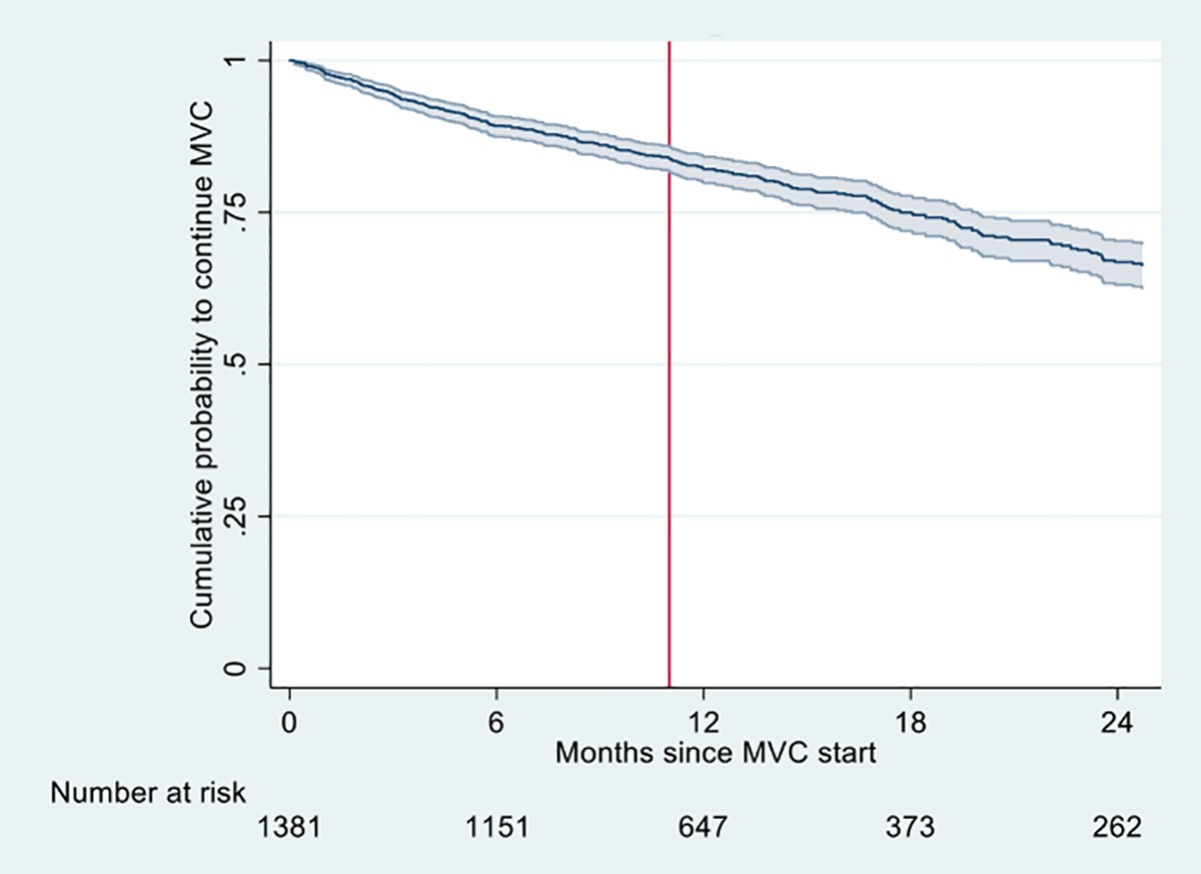
Kaplan-Meier curve of the cumulative probability of continuing maraviroc after its initiation in the study cohort. The line indicates the estimate, shadows represent the 95% confidence intervals. MVC, maraviroc. The number of patients receiving maraviroc are indicated as “Number at risk”. The vertical line shows the 48 weeks cut-off point; all available data up to 24 months of follow-up were used.

Duration of follow-up and number of visits differed from centre to centre because of varying clinical practice. Several sites provided only data covering 48 weeks after maraviroc initiation, whereas others reported all available follow-up. During 1,845 person-years of follow-up, maraviroc was discontinued in 354 patients. The survival analysis (Fig 1) showed a stable rate of maraviroc discontinuation during the first 2 years, with a 1-year estimated probability of continuing maraviroc of 82.1% (95% CI, 79.9–84.2). Durability was estimated based on patients continuing to receive maraviroc, regardless of VF. Reasons for maraviroc discontinuation were reported for 294 patients and comprised VF (92/294, 31.4%), treatment simplification (27/294, 9.3%), clinician’s decision (29/294, 9.8%), toxicity (53/294, 18.1%), non-adherence (37/294, 12.7%), patient’s choice (10/294, 3.4%), non-R5 tropism (6/294, 2.0%), loss to follow-up (6/294, 2.0%), death (6/294, 2.0%), and other reasons (27/294, 9.3%). Reported toxicity reasons comprised two cases of serum transaminases elevation.

Overall, 572/1,381 (41.4%, 95% CI: 38.9–44.1) subjects experienced TF over 48 weeks of follow-up. The logistic regression analysis of factors associated with TF is shown in Table 4. After adjustment, the odds of TF were increased by a low nadir CD4 count, a detectable baseline viral load, having previously experienced PI therapy, a non-R5 tropism result, a GSS of the regimen accompanying maraviroc ≥3 and starting maraviroc after 2011. There was also a marginal effect of age.

**Table 4:** Univariate and multivariable logistic regression analysis of factors associated with treatment failure over 48 weeks.

		Univariate analysis			Multivariablee analysis		
		N	Failure (%)	p-value	AOR	95% CI	p-value
**Age (years)**	<35	151	50.3	0.04	1.00		0.05
	≥35	1212	40.1		0.68^a^	0.46–0.99	
	35–44	458	38.0				
	45–54	532	42.7				
	≥55	222	38.3				
**Ethnicity**	White Caucasian	718	40.8	0.17	NI		
	Black African	231	42.9				
	Asian	18	33.3				
	Other	29	51.7				
**Nadir CD4 count (cells/mm**^**3**^**)**	<50	285	51.9	<0.01	1.00		
	50–199	469	41.4		0.70	0.50–0.97	0.03
	200–349	290	39.7		0.73	0.51–1.05	0.09
	≥350	148	35.1		0.48	0.30–0.77	<0.01
**Baseline CD4 count (cells/mm**^**3**^**)**	<50	51	58.8	<0.01	NI		
	50–199	236	47.9				
	200–349	257	39.3				
	≥350	782	37.7				
**Baseline HIV-1 RNA (cps/mL)**	<50	630	32.5	<0.01	1.00		
	50–199	147	48.3		1.82	1.01–3.27	0.04
	200–4,999	253	51.4		2.45	1.46–4.11	<0.01
	5.000–99,999	189	39.7		1.80	1.02–3.33	0.04
	≥100,000	120	54.2		2.98	1.67–6.03	<0.01
**Drugs experienced (n)**	<3	329	36.5	0.11	NI		
	3–6	345	43.8				
	7–10	320	47.8				
	≥10	333	39.9				
**ART classes experienced, n **	1	455	36.9	0.01	NI		
	2–3	926	43.6				
**Maraviroc daily dose**	≥300 mg	167	50.9	<0.01	0.72	0.49–1.06	0.09
	<300 mg	992	38.8		1.00		
**Maraviroc schedule**	Once daily	276	46.0	<0.01	NI		
	Twice daily	661	38.6				
**Context of maraviroc use**	Naive	46	41.3	<0.01	0.48	0.19–1.20	0.12
	Switch, suppressed VL	409	32.3		1.00		
	Switch, detectable VL	441	49.0		1.21	0.68–2.16	0.51
	Intensify, suppressed VL	50	42.0		1.52	0.79–2.94	0.21
	Intensify, detectable VL	50	56.0		1.42	0.63–3.23	0.40
**Previous PI experience**	Yes	1218	42.9	0.04	1.62	1.05–2.48	0.03
	No	163	30,7		1.00		
**Viral tropism**	R5	1061	39.1	<0.01	1.00		
	Non-R5	165	50.3		1.65	1.20–2.27	<0.01
**FPR**	≤10	51	51.0	<0.01	NI		
	(10–20]	68	48.9				
	(20–40]	156	37.3				
	≥40	330	40.0				
**GSS of accompanying regimen**	<1	83	27.7	<0.01	1.00		
	1–2	254	37.8		1.33	0.64–2.76	0.34
	2–3	228	32.9		1.08	0.52–2.26	0.69
	≥3	96	55.2		2.47	1.07–5.66	0.01
**Year of maraviroc start**	≥2012	681	60.9	<0.01	3.20	2.39–4.30	<0.01
	<2012	127	37.3		1.00		

Note: Table refers to 1,381 subjects; cases with missing values for each variable are not reported in the table; in 264 cases viral tropism was collected but no FPR was available at the data analysis

NI: Not included in the final model

In the univariate analysis only variables with p-value <0.20 are shown. Additional variables tested but not found to be associated (p ≥0.20) in univariate analysis comprised: gender, previous experience with NRTI, NNRTI, integrase inhibitors or enfuvirtide, and viral subtype.

^a^(≥ 35 vs <35 years)

All variables shown in the univariate analysis were included in the multivariable model. Variables retained in the final step of the backward elimination procedure (see methods) are shown, all the other were excluded, except the viral tropism result than was manually included. All AOR shown are simultaneously adjusted. The FPR of the geno2pheno[coreceptor] genotypic tropism interpretation was not included in the multivariable model due to co-linearity with tropism result.

Of the 1,010 patients still on maraviroc at week 48, 201 (19.9%) had a viral load >50 copies/mL, including 93 (9.2%) with levels between 50 and 200 copies/mL and 108 (10.7%) with levels >200 copies/mL. The logistic regression analysis of factors associated with VF is shown in Table 5. After adjustment, the odds of VF were increased by a low nadir CD4 count, a detectable baseline viral load, and having previously experienced PI therapy. There was also a marginal effect of age.

**Table 5:** Univariate and multivariable logistic regression analysis of factors associated with virological failure at 48 weeks.

		Univariate analysis			Multivariable analysis		
	* *	N	Failure (%)	p-value	AOR	95% CI	p-value
Age (years)	<35	105	28.6	0.19	1.00		0.05
	≥35	897	19.1		0.58^a^	0.35–0.95	
	35–44	354	20.0				
	45–54	379	19.3				
	≥55	164	16.5				
Nadir CD4 count (cells/mm^3^)	<50	195	29.7	<0.01	1.00		
	50–199	335	17.9		0.55	0.35–0.85	<0.01
	200–349	209	16.3		0.57	0.34–0.94	0.03
	≥350	112	14.3		0.42	0.22–0.81	<0.01
Baseline CD4 count (cells/mm^3^)	<50	31	32.3	<0.01	NI		
	50–199	170	27.6				
	200–349	196	20.4				
	≥350	584	16.6				
Baseline HIV-1 RNA (cps/mL)	<50	473	10.1	<0.01	1.00		
	50–199	110	30.9		3.59	2.13–6.03	<0.01
	200–4.999	182	32.4		4.45	2.83–6.98	<0.01
	5.000–99.999	141	18.6		2.37	1.39–4.03	<0.01
	≥100.000	81	32.9		4.88	2.74–8.69	<0.01
Maraviroc daily dose	<300 mg	105	21.9	0.33	NI		
	≥300 mg	752	19.3				
Maraviroc schedule	Once daily	190	21.6	0.52	NI		
	Twice daily	498	18.5				
Context of maraviroc use	ART naive	32	15.6	<0.01	NI		
	Switch. suppressed VL	303	8.6				
	Switch. detectable VL	310	27.7				
	Intensify. suppressed VL	34	14.7				
	Intensify. detectable VL	37	40.5				
Drug classes previously experienced	<3	361	17.2	0.13			
	3	337	19.9				
	≥3	311	23.5				
Previous PI experience	Yes	876	21.0	0.05	2.21	1.21–4.04	0.01
	No	133	13.7				
Previous INSTI experience	Yes	*456*	22.4	0.07	NI	* *	* *
	No	*553*	17.9			* *	* *
Previous enfuvirtide experience	Yes	*61*	26.2	0.18	NI	* *	* *
	No	*948*	19.5			* *	* *
Viral tropism	R5	805	19.8	0.06	1.00		
	Non-R5	108	25.2		1.25	0.79–1.96	0.34
ART classes used with maraviroc (n)	1	*378*	15.9	0.02	NI	* *	* *
	2	*457*	21.0			* *	* *
	3	*105*	28.6			* *	* *
Concomitant PI use	Yes	*655*	21.3	0.17		* *	* *
	No	*285*	16.1			* *	* *
GSS of accompanying regimen	<1	73	17.8	<0.01	1.00		
	1 <2	191	17.3		0.82	0.64–2.76	0.61
	2 <3	188	18.6		0.86	0.40–1.85	0.69
	≥3	65	33.8		2.14	0.90–5.09	0.08

Note: Table refers to 1009 subjects; cases with missing values for each variable are not reported in the table; in 264 cases viral tropism was collected but no FPR was available at the data analysis. Additional variables tested but not found to be associated by univariate analysis (p>0.2): maraviroc schedule and daily dosing, gender, ethnicity, number of previous drugs experienced, previous experience with NRTIs, or NNRTIs, viral subtype, false positive rate value of the geno2pheno[coreceptor] genotypic tropism interpretation, and calendar year of maraviroc start.

^a^(≥ 35 vs <35 years)

All variables shown in the univariate analysis were included in the multivariable model. Variables retained in the final step of the backward elimination procedure (see methods) are shown. all the other were excluded, except the viral tropism result that was manually included. All AOR shown are simultaneously adjusted.

Tables 6 and 7 show the outcomes for the relatively high number of subjects that received maraviroc outside of standard treatment guidelines. Overall, TF was more likely in subjects receiving maraviroc once daily, those receiving a total daily dose of 150mg and those with non-R5 tropism (Table 6), whereas only non-R5 tropism showed a marginal effect on the occurrence of VF (Table 7).

**Table 6:** Univariate logistic regression analysis of the association between non-standard maraviroc uses and treatment failure.

		Treatment failure over 48 weeks						
	* *	No		Yes		Total	p-value*	p-value**
	* *	N	%	N	%			
Dosing schedule	Once daily	149	54.0	127	46.0	276	<0.01	0.01
	Twice daily	406	61.4	255	38.6	661		
	Unknown	254	57.2	190	42.8	444		
								
Total daily dose (mg)	150	82	49.1	85	50.9	167	<0.01	0.01
	300	434	59.2	299	40.8	733		
	600	153	67.1	75	32.9	228		
	1200	20	64.5	11	35.5	31		
	≥150	607	61.2	385	38.8	992		
	Unknown	120	54.1	102	45.9	222		
								
Tropism	R5	643	61.3	406	38.7	1049	<0.01	<0.01
	Non-R5	107	47.8	117	52.2	224		
	Unknown	58	53.7	50	46.3	108		

Note: all p-values were calculated by a univariate mixed logistic model that takes into account the clustering effect of the clinical centre

* p-value including unknown category

** p-value excluding unknown category

**Table 7:** Univariate logistic regression analysis of the association between non-standard maraviroc uses and virological failure.

		Virological failure (week 48)						
	* *	No		Yes		Total	p-value*	p-value**
	* *	N	%	N	%			
Dosing schedule	Once daily	149	78.4	41	21.6	190	0.52	0.36
	Twice daily	406	81.5	92	18.5	498		
	Unknown	254	78.9	68	21.1	322		
								
Total daily dose (mg)	150	82	78.1	23	21.9	105	0.45	0.33
	300	434	79.2	114	20.8	548		
	600	153	84.5	28	15.5	181		
	1200	20	87.0	3	13.0	23		
	≥150	607	80.7	145	19.3	752		
	Unknown	120	78.4	33	21.6	153		
								
Tropism	R5	643	80.2	159	19.8	802	0.06	0.15
	Non-R5	107	74.8	36	25.2	143		
	Unknown	59	90.8	6	9.2	65		

Note: all p-values were calculated by a univariate mixed logistic model that takes into account the clustering effect of the clinical centre

* p-value including unknown category

** p-value excluding unknown category

## Discussion

This study reports the largest data set of maraviroc-containing treatment cases ever collected from routine clinical practice. We asked clinical sites to include all their patients initiating maraviroc outside clinical trials to assess the use and effectiveness of maraviroc across Europe, in a time frame spanning from 2005 to 2016. Based on this survey enrolling cases through 26 different clinical sites in 8 European countries, we observed that maraviroc was used in multiple clinical scenarios. Its most frequent use was as a switch therapy in highly ART-experienced patients with either detectable or undetectable viral load. In this population, maraviroc use was safe and reasonably effective. Maraviroc discontinuation was mostly due to VF, desire to simplify the treatment regimen, or issues of incomplete adherence with the regimen. Toxicity accounted for 18% of the reported reasons of maraviroc discontinuation. Clearly, neither toxicity nor VF could be specifically attributed to maraviroc, due to concomitant antiretrovirals. This being a retrospective study, it was not possible to investigate the details of the toxicity events and confirm their attribution to maraviroc or other drugs. Notably, only two cases of transaminase elevations were reported as reasons for maraviroc discontinuation. Major determinants of treatment failure–defined as discontinuation of maraviroc for any reason or virological failure–were the concomitant use of three or more predicted active drugs, and more recent calendar year of maraviroc initiation (after 2011). These findings are likely to reflect a higher propensity for maraviroc interruption in the presence of other active drugs, and the increased number of effective drugs that have become available in recent years.

Interestingly, a significant proportion (17.6%) of patients in the cohort started maraviroc despite carrying a non-R5 virus. Given that the classification as R5 was largely based on the centralised re-analysis of V3 sequences, the observed off-label use of maraviroc may in part reflect technical aspects of tropism testing and the application of different interpretation thresholds. There also appeared to be interest in exploring maraviroc activity in patients with mixed tropism results and in those for whom selective tissue activity was postulated (i.e., against virus replicating in the CNS– 5 cases indeed reported this reason for maraviroc use). Non-R5 tropism predicted higher odds of treatment failure, however it had a weaker effect on virological failure, with a higher proportion of patients with non-R5 virus experiencing virological failure in a univariable analysis but no clear association in the adjusted model. This suggests that some of the non-standard use might have been explorative and short-term. In this context, tropism would not be expected to be the key predictor of virological failure. In addition, in the highly treatment-experienced population we analysed, multiple determinants are expected to impact virologic responses. There is evidence from the MOTIVATE trials of maraviroc in treatment-experienced patients that the composition of the overall regimen (alongside the baseline CD4 count) is a key predictor of virologic outcome, including in a small subset of patients with non-R5 virus [20].

About one in five patients on maraviroc had a detectable viral load after 48 weeks of follow-up. It should be noted that the analysis of virological failure excluded patients who either previously discontinued or were lost to follow-up, and that these may have comprised cases of virological failure. Although this may be considered a high rate of virological failure (when defined as a viral load >50 copies/mL), interpretation should consider the high stringency applied to the definition of failure. Indeed, applying a higher and clinically more relevant viral load threshold of 200 copies/mL halved the number of virological failures, indicating that a substantial number of cases of viraemia were at low level. Moreover, the cohort had a significant history of treatment exposure with about two thirds of participants having previously experienced 3 or more antiretroviral drug classes. Predictors of virological failure were a low nadir CD4 cell count, a detectable viral load at maraviroc initiation, and a history of PI exposure, with a marginal effect of younger age. In line with these findings, previous cohort studies showed that older age reduces the risk of virological failure, likely as a result of improved medication adherence [21]. Higher baseline viral load and lower nadir CD4 counts were also associated with virological failure in other observational studies [22–24]. It should be noted that a low nadir CD4 count may indicate the presence minority CXCR4-tropic variants which would remain undetected by routine testing methods [25,26]. The association of higher risk of virological failure with prior PI use may reflect circumstances associated with increased PI use including treatment failure, drug resistance and low medication adherence. Interestingly, we found no association between the activity of the regimen accompanying maraviroc (measured as the GSS) and virological failure. This implies that non-resistance related factors largely accounted for virological outcomes. For example, pre-treated patients with high GSS values may actually reflect past failures due to non-adherence to therapy. On the other hand, in a population with extensive treatment experience, the extent of archived drug resistance and residual drug activity despite resistance may both be underestimated [27].

The treatment collection included several cases of non-standard, off-label maraviroc use. Indeed, it included ART-naïve subjects (unlicensed indication in Europe), patient carrying non-R5 virus, and cases with once-daily maraviroc dosing and low daily doses of 150mg. While we cannot exclude prescription errors, it is also realistic to assume that the drug was deliberately used in off-label indications. Off-label use may result from lack of alternative treatment options because of toxicity or drug resistance, hypothesised residual drug activity [28], or the need for dosing simplification to once-daily regimens and lower drug dosing as suggested by pharmacokinetics reports, in association with boosted PI [29]. Overall, we found evidence that using maraviroc either once daily, at a low total daily dose of 150mg, or with non-R5 virus increased the likelihood of treatment failure, but the effect on virological failure was only marginally apparent with non-R5 virus. One relatively common use of maraviroc was treatment intensification due to low CD4 cell counts. This reflects prior evidence of an immunologic benefit of maraviroc use, independent from its antiviral efficacy, which was not confirmed by subsequent controlled studies [30,31].

This study has several limitations. First, no measure of medication adherence was available, therefore this major determinant of treatment outcome could not be analysed. This is not different from the majority of other reported observational studies of ART outcomes, which did not collect medication adherence systematically and could not adjust for this factor. Second, some variables such as genotypic susceptibility of the concomitant regimen and viral subtype were not available for a large proportion of cases, therefore their predictive value could not be fully analysed. Third, the retrospective design of the study did not allow to confirm the reasons for maraviroc initiation or interruption. In order to partly overcome this limitation, we used objective data such as the pre-treatment viral load, the pre-maraviroc treatment regimen, and the drugs prescribed concomitantly with maraviroc to classify the different clinical scenarios of maraviroc prescription. Despite these limitations, this study is unique in depicting the clinical use of maraviroc in clinical practice throughout Western Europe during the last decade.

In conclusion, in this large European survey we found that maraviroc was used in multiple clinical scenarios involving mostly ART-experienced patients, both virologically suppressed and with detectable viremia. In a population of highly experienced patients, maraviroc was overall safe and reasonably effective, also when considering that there was a considerable use of exploratory off label treatments, and a substantial number of virological failures occurred at viral load <200 copies/mL. High discontinuation rates in recent years presumably reflected the availability of alternative active agents. In current guidelines, maraviroc continues to represent a valid antiretroviral option in selected patients, particularly those with limited drug options or intolerance to agents from other classes. The virological rationale for using CCR5 antagonists early in therapy is still strong, as reflected by the significant impact of a low nadir CD4 count on treatment outcomes [32]. Whether CCR5 antagonists exhibit additional benefits beyond their antiviral activity remains to be established [33–35].
